# Development and Validation of a 12-Gene Immune Relevant Prognostic Signature for Lung Adenocarcinoma Through Machine Learning Strategies

**DOI:** 10.3389/fonc.2020.00835

**Published:** 2020-05-27

**Authors:** Liang Xue, Guoshu Bi, Cheng Zhan, Yi Zhang, Yunfeng Yuan, Hong Fan

**Affiliations:** Department of Thoracic Surgery, Zhongshan Hospital, Fudan University, Shanghai, China

**Keywords:** lung adenocarcinoma, risk score formula, immune infiltration, machine learning, survival

## Abstract

**Background:** Although immunotherapy with checkpoint inhibitors is changing the face of lung adenocarcinoma (LUAD) treatments, only limited patients could benefit from it. Therefore, we aimed to develop an immune-relevant-gene-based signature to predict LUAD patients' prognosis and to characterize their tumor microenvironment thus guiding therapeutic strategy.

**Methods and Materials:** Gene expression data of LUAD patients from Gene Expression Omnibus (GEO) and The Cancer Genome Atlas (TCGA) were systematically analyzed. We performed Cox regression and random survival forest algorithm to identify immune-relevant genes with potential prognostic value. A risk score formula was then established by integrating these selected genes and patients were classified into high- and low-risk score group. Differentially expressed genes, infiltration level of immune cells, and several immune-associated molecules were further compared across the two groups.

**Results:** Nine hundred and fifty-four LUAD patients were enrolled in this study. After implementing the 2-steps machine learning screening methods, 12 immune-relevant genes were finally selected into the risk-score formula and the patients in high-risk group had significantly worse overall survival (HR = 10.6, 95%CI = 3.21–34.95, *P* < 0.001). We also found the distinct immune infiltration patterns in the two groups that several immune cells like cytotoxic cells and immune checkpoint molecules were significantly enriched and upregulated in patients from the high-risk group. These findings were further validated in two independent LUAD cohorts.

**Conclusion:** Our risk score formula could serve as a powerful and accurate tool for predicting survival of LUAD patients and may facilitate clinicians to choose the optimal therapeutic regimen more precisely.

## Background

Lung cancer is the most common cancer worldwide and the leading cause of cancer death in men and women (18.4% of the total cancer deaths), accounting for an estimated 2,093,876 new cases (11.6% of the total cases) in 2015 (1). The 5-year survival rate of lung cancer is still low, which was 21.2% in the USA and 19.8% in China (2). Followed by lung squamous cell carcinoma, lung adenocarcinoma (LUAD) is currently the most common subtype of non-small cell lung cancer (NSCLC), which accounts for more than 40% of lung cancer incidence (3). Complete surgical resection remains the standard treatment method for early LUAD, while adjuvant or neoadjuvant therapy including chemotherapy and radiotherapy are given to selected patients. Up to now, risk factors such as TNM stage and age are commonly used for predicting LUAD patients' survival and determining therapeutic regimen. However, LUAD within the same TNM stage might also have different prognosis because of the inherent clinicopathological and molecular diversities. Therefore, numerous prognostic models integrating clinical factors and gene expression data have been provided as a supplement to traditional TNM staging system (4–6). However, most of these studies failed to take the biological functions of prognostic genes into account before the gene selection process.

In the past decade, immune checkpoint blockade such as Nivolumab and Pembrolizumab has delivered unprecedented success in treating NSCLC to extend overall survival (7–9). However, only a small percentage of patients experience such clinical benefits (10). Therefore, a multi-immune-relevant-gene-based signature which enables clinicians to predict LUAD patients' prognosis and to characterize their tumor microenvironment is urgently demanded.

To address this problem, in this study we performed Cox regression analysis and random survival forest algorithm to identify the immune relevant genes with potential prognostic value from an immune gene list. A risk-score system was constructed to predict patients' risk in both discovery and validation cohorts, and the immune infiltration patterns of patients with different risk score were comprehensively depicted. We believe that our gene signature and corresponding risk score will facilitate clinicians to predict LUAD patients' prognosis and choose the optimal treatment more precisely.

## Methods and Materials

### LUAD Datasets Acquisition and Preprocessing

For (Gene Expression Omnibus) GEO data, the criteria for enrollment of public available LUAD patient's data was as follows: the gene expression data was generated by the same chip platform (Affymetrix Human Genome U133 Plus 2.0 chips) and reliable clinical survival information were accessible. After systematically screening, microarray data from GSE31210, GSE41271, and GSE50081 datasets representing different independent studies of LUAD were directly downloaded from GEO (http://www.ncbi.nlm.nih.gov/geo). The probe sets of Affymetrix Human Genome U133 Plus 2.0 chips were annotated to gene names based on the annotation platform GPL570, while the list of immune-relevant genes was obtained from https://www.immport.org/shared/home (*n* = 1811). The batch effect resulting from the heterogeneity among different microarray data sets were eliminated by the use of *sva* package (11), while the background adjustments and data normalization were performed with *limma* package (12).

As for TCGA (The Cancer Genome Atlas) data, the LUAD legacy level-3 RNA sequencing data were downloaded and normalized using the *TCGAbiolinks* R package (13). Corresponding baseline demographic and clinical information were acquired from UCSC Xena Database (http://xena.ucsc.edu/). We removed the patients whose clinical outcome information including survival time and vital status were vague or absent. The pathological stages of the patients included in this study were updated according to the 7th edition of the American Joint Committee on Cancer criteria.

### Identification of Potential Genes Using Bioinformatics Dimension Reduction Algorithm

We downloaded the list of 1,881 immune relevant genes from Immport Database (https://www.immport.org) (14). Cox regression proportional hazards regression analysis was employed for the primary screening from the 1,881 immune relevant genes for potential prognostic ones. Each gene was analyzed as an independent overall survival (OS)-related prognostic variable by multivariable analysis with the adjustments of age, gender, TNM stage, and smoking status. In the present study, the independent hazard ratio (HR) and corresponding 95% confidence interval for each gene was calculated by the implementation of *survival* package. The genes whose *p*-value was <0.05 were considered as significant prognostic genes.

The random forest algorithm, a machine learning dimension reduction strategy based on the construction of thousands of classification or regression trees, has been widely used in variable selection of high-dimensional data, while the *randomForestSRC* package makes it possible for researchers to analyze survival data with this method (15). As suggested by Ishwaran et al., we set the number of nsplit at 10 in the variable hunting function (16, 17). Genes were further selected out if their VIMP (variable importance), which measures the variation of the random forest model's prediction error rate when a gene was randomly added in the model, was higher than 0.01.

After this two-step filtration method, a risk score formula was then established by including each of these selected genes, weighted by their estimated regression coefficients in the Cox regression analysis (18), as follows:

(1) $$\begin{array}{r} \text{Risk Score}=\overset{N}{\underset{i=1}{\sum }}\beta_{i}^{*}\left(Expression\;level\;of\;Gene_{i}\right) \end{array}$$

Where *N* represents the number of finally enrolled genes, β indicates the coefficient of *Gene*_*i*_ obtained from the first-step Cox regression analysis. The risk score of each patient included in this study was calculated by this formula, with which patients were assigned into high- or low-risk group by using the corresponding median risk score as the cutoff value. The heatmaps and clustering analyses were generated by the use of *pheatmap* package.

### Comparison of Enriched Oncogenic Pathways

Identification of differentially expressed genes (DEGs) between the high- and low-risk groups was conducted using package *limma* (12). Fold change > 1.0 and adjusted *P* < 0.05 were considered as the cutoff criteria to screen for DEGs. Functional enrichment analyses on the detected DEGs were performed with the *clusterProfiler* package (19). Gene ontology (GO) terms were identified with a strict cutoff of adjusted *P* < 0.01 and false discovery rate (FDR) <0.05. Meanwhile, to explore the enrichment pattern of other relevant biological processes, we employed gene signatures proposed by Liberzon et al. for single sample gene set enrichment analysis (ssGSEA, from *GSVA* package) (20, 21).

### Estimation of Immune Cell Abundance by ssGSEA

To construct a compendium of microenvironment genes related to specific microenvironment cell subsets, we systematically searched the published papers and combined the gene signatures respectively proposed by Angelova et al. (22), Newman et al. (23), and Becht et al. (24), which consisted of 384 genes representing 28 microenvironment cell subsets from both innate and adaptive immunity, including T cells, eosinophils, mast cells, endothelial, dendritic cells, B cells, macrophages, NK cells, MDSC, neutrophils, monocytes, and fibroblasts. Subsequently, we used ssGSEA in *GSVA* package based on deconvolution algorithm to estimate the relative infiltration level of each cell population in each LUAD sample with expression data. Several other immune associated factors comparison between two groups, including tumor purity, leukocyte fraction, TGF response, INF-gamma response, cytotoxic cell fraction, PDCD1, CD274, CTLA4 expression differences, were also performed as previously reported (9, 25–27).

### Statistical Analysis

All statistical analyses were conducted using R software (Version 3.5.3; R Foundation for Statistical Computing, Vienna, Austria) (28, 29) and Stata (Version 13.0, Stata Corp, College Station, TX, USA). A description and comparison of the baseline characteristics of the patients from different risk groups was conducted in which categorical variables were compared by the chi-square test and Fisher's exact test when appropriate. Kaplan–Meier survival curves visualized by *ggplot2* package and log-rank tests were used to compare the OS and between different populations. Receiver operating characteristic (ROC) analyses were conducted to evaluate the sensitivity and specificity of the survival predicting model based on the risk score and other clinical factors. In the chi-square test, Fisher's exact test and log-rank test, the *P* < 0.05 was considered as significant.

## Results

### LUAD Patients' Data Preparation and Description

**Figure 1:**
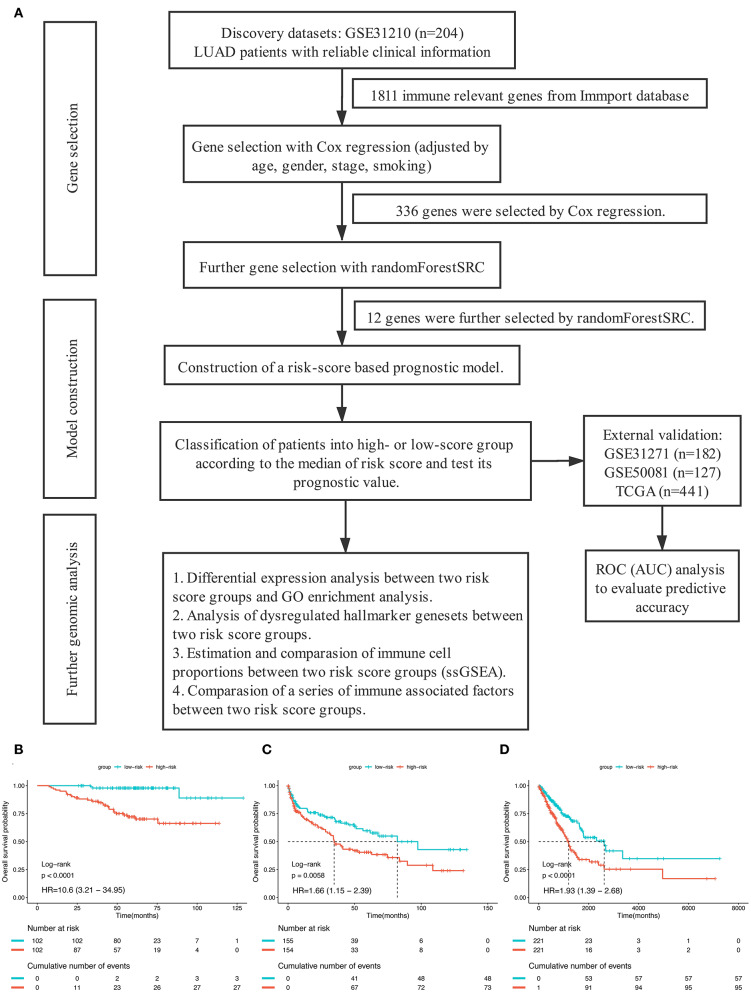
**(A)** The overall design of this study. **(B–D)** Kaplan–Meier curves for overall survival (OS) stratified by risk-group in GSE31210 cohort **(B)**, GSE41271 and GSE50081 cohort **(C)**, and TCGA cohort **(D)**.

The overall study design was shown in Figure 1A. After systematically searching for LUAD gene expression data and corresponding clinical information that were publicly available, a total of 954 patients from four independent LUAD cohort were finally enrolled in the present study: GSE31210 (*n* = 204), GSE41271 (*n* = 182), GSE50081 (*n* = 127), and TCGA (*n* = 441). We assigned the 204 patients in GSE31210 to the discovery group, 309 patients in GSE41271, and GSE50081 to the GEO external validation group and the remained 441 patients to the TCGA external validation group. The median overall survival time of patients in the discovery group, GEO external validation group, and TCGA validation group were, respectively, 60.5 months (range from 7.4 to 128.8), 45.1 months (range from 0.3 to 134.2), and 20.85 months (range from 4 to 241.6). The clinical data of the patients enrolled were provided in Tables S1–S3.

### Identification of Prognostic Genes and Construction of the Risk-Score System

We employed different algorithms, including multivariable Cox and random survival forest, to identify prognostic genes from the 1,811 immune-relevant gene list in the discovery dataset. First, by fitting the expression data in GSE31210 into multivariable Cox regression proportional hazards regression analysis one-by-one in combination with the adjustments of age, gender, TNM stage and smoking, the optimal cut-off value for each gene's expression level were, respectively, determined by the use of “*Survminer*” packages and the corresponding HR and *p*-value were also computed. Basing on the result of Cox analysis, we primarily detected 336 significant genes whose *p*-value were <0.05 (Table S4). The 336 genes were then analyzed by random survival forest-variable hunting (RSF-VH) algorithm for further screening. Finally, 12 gene were selected out as variable importance was larger than 0.01. The permutation *p*-values, HR, coefficient from the univariable analysis, and the corresponding VIMP were shown in Table S5.

**Figure 2:**
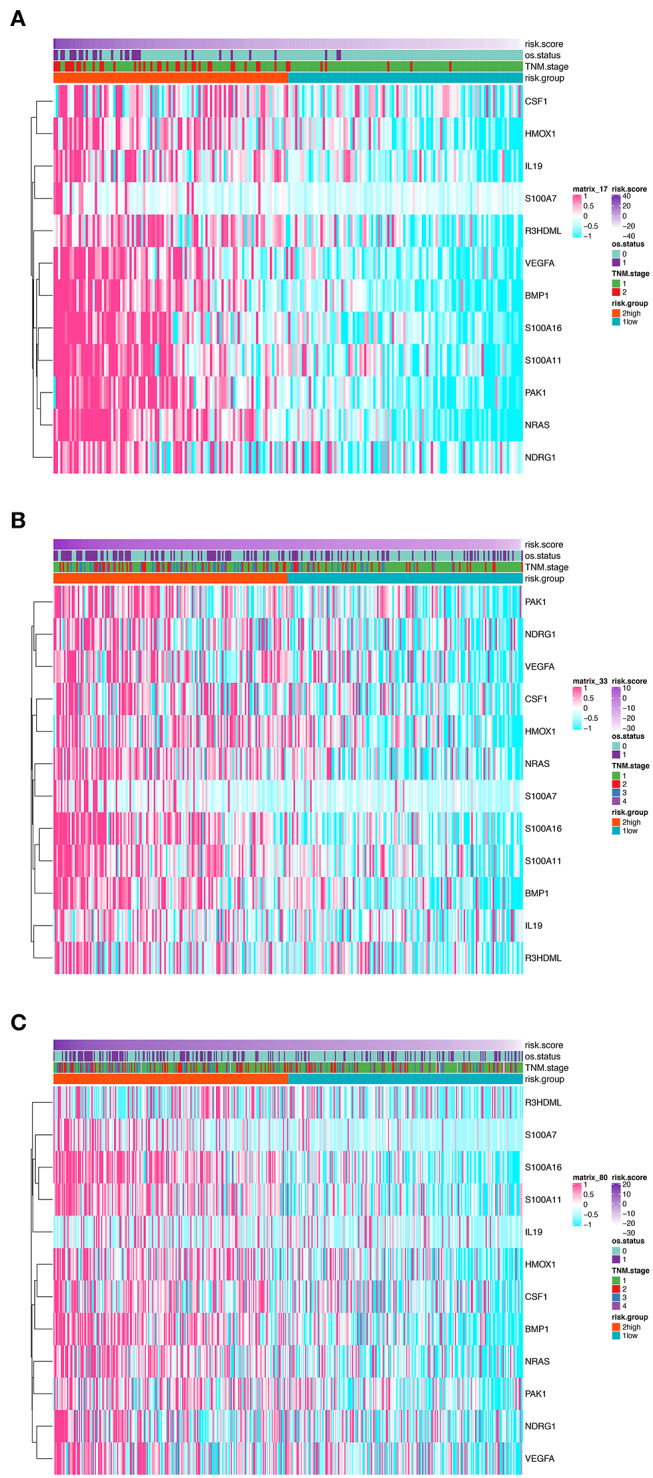
Heatmaps presenting the relative expression level of the 12-gene signature and corresponding risk score in patients from GSE31210 cohort **(A)**, GSE41271 and GSE50081 cohort **(B)**, and TCGA cohort **(C)**. Clinicopathological information including TNM stage, vital status, as well as risk group, are shown in annotations above.

In order to establish a clinically applicable risk assessment model for different populations of LUAD patients, a risk-score system was built based on the expression of these 12 genes and corresponding coefficient generated from the univariable Cox regression analysis. The formula is as follows: Risk score = (1.009^*^ expression level of S100A7) + (1.482^*^ expression level of R3HDML) + (1.312^*^ expression level of IL19) + (2.409^*^ expression level of NRAS) + (1.764^*^ expression level of BMP1) + (0.887^*^ expression level of S100A11) + (1.494^*^ expression level of HMOX1) + (1.969^*^ expression level of PAK1) + (2.292^*^ expression level of S100A16) + (1.563^*^ expression level of VEGFA) + (1.088^*^ expression level of NDRG1) + (1.115^*^ expression level of CSF1). We then computed the risk-score for each patient in the discovery group and set the median of the risk score (−2.65) as optimal cutoff value to classify cases into high risk and low-risk groups. The baseline clinical and pathological information of patients from the two groups were summarized in Table 1 (102 patients in high-risk group and 102 in low-risk group, data available in Table S1). As shown in Figure 1B, the Kaplan–Meier survival demonstrated that the patients in high-risk group had significantly worse OS than those in low-risk group in the discovery dataset (HR = 10.6, 95%CI = 3.21–34.95, log-rank *P* < 0.001). The distribution of the expression level of these 12 genes among the patients, and corresponding risk score, risk group, vital status, and TNM stage were shown in Figure 2A, which displayed the all-round relative enrichment of these genes in high-risk group, indicating that the 12 immune relevant genes were all associated with worse survival.

**Table 1:** The baseline clinical and pathological information of patients from the two groups in the discovery cohort.

**Variables**	**Total (*n* = 204)**	**Low-risk (*n* = 102)**	**High-risk (*n* = 102)**	***P*-value**
Age	61 (55–63)	I61 (56–63)	60 (54–63)	0.389
**Sex**				
Female	109 (53%)	63 (62%)	46 (45%)	0.025
Male	95 (47%)	39 (38%)	56 (55%)	
**Smoking**				
No	105 (51%)	63 (62%)	42 (41%)	0.005
Yes	99 (49%)	39 (38%)	60 (59%)	
**Pathological stage**				
Stage I	162 (79%)	96 (94%)	66 (65%)	<0.001
Stage II	42 (21%)	6 (6%)	36 (35%)	<0.001

**Figure 3:**
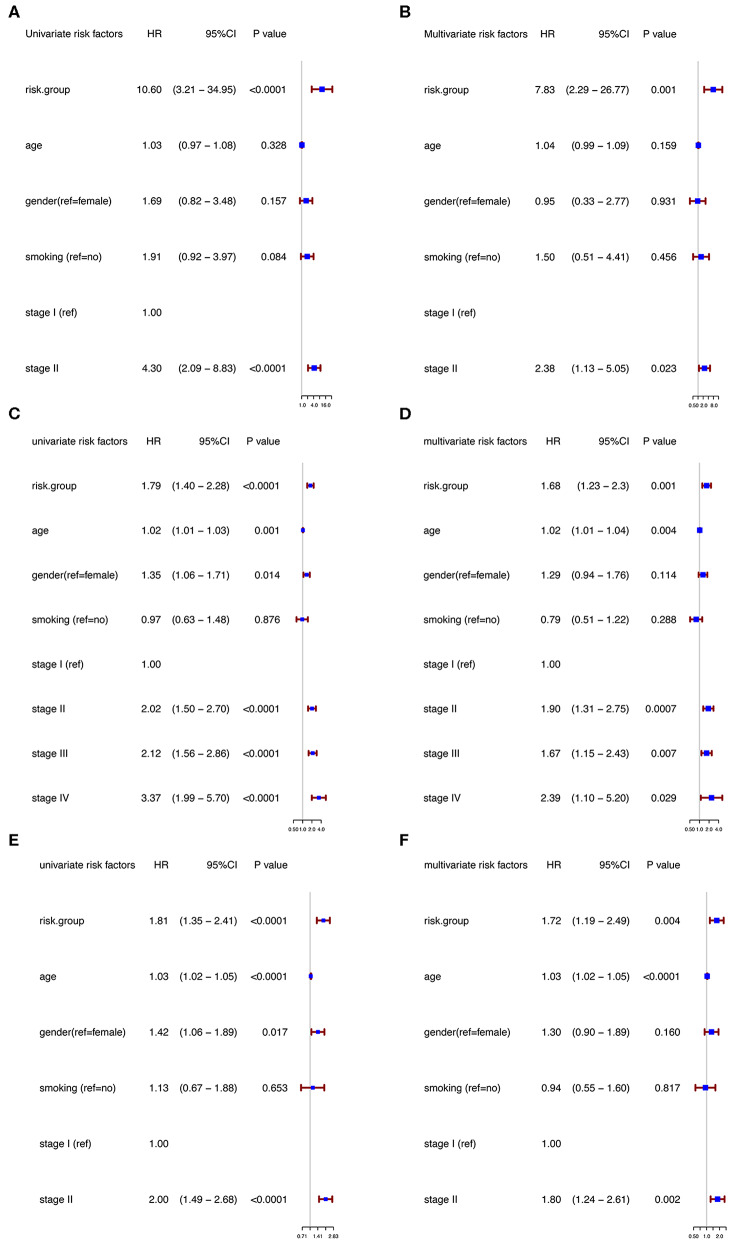
Univariable and multivariable analyses based on risk group and other clinical factors in GSE31210 cohort [Univariable **(A)**, Multivariable **(B)**], GSE41271 and GSE50081 cohort [Univariable **(C)**, Multivariable **(D)**], and TCGA cohort [Univariable **(E)**, Multivariable **(F)**].

Moreover, in the univariable Cox regression analyses, the risk group was a strong variable correlated with worse prognosis (Figure 3A). After multivariable adjustment by other clinical factors including age, gender, smoking, and TNM stage, the risk group remained a significant and independent prognostic indicator in the discovery group (Figure 3B).

**Figure 4:**
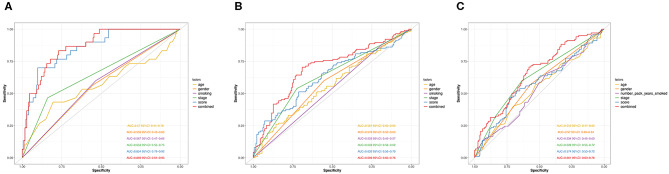
ROC curves measuring the predictive value of the risk score and other clinical factors in GSE31210 cohort **(A)**, GSE41271 and GSE50081 cohort **(B)**, and TCGA cohort **(C)**.

Next, we performed ROC analysis to assess the sensitivity and specificity of the risk score system, age, gender, smoking, and TNM stage. The area under receiver operating characteristic (AUCROC) for 5-year OS was calculated to comprehensively depict the prognostic accuracy of these factors and the combined formula. As shown in Figure 4A, the AUCROC of the 12-genes risk score (blue) (AUCROC = 0.854, 95%CI = 0.79–0.92) was significantly superior than that of other variables (AUCROC = 0.57, 0.559, 0.567, and 0.653 for age, gender, smoking, and stage, respectively, all *P* < 0.01). Additionally, when combining all these factors together, the model with the strongest power for OS predictive ability could be achieved (AUCROC = 0.869, 95%CI = 0.81–0.93).

### Assessing the Performance of the 12-Genes Model in GEO and TCGA Validation Cohorts

To further evaluate the robustness of the risk score system based on the 12 immune relevant genes, we performed similar analyses in the GEO and TCGA external validation cohorts using the median score of the validation cohorts as the cut-off value (high score group: *n* = 154 in GEO and *n* = 221 in TCGA; low score group: *n* = 155 in GEO and *n* = 221 in TCGA). In consistence with the findings mentioned above, high risk score group was significantly associated with worse survival outcomes in both of the two validation cohorts (in GEO: HR = 1.66, 95%CI = 1.15–2.39, log-rank *P* = 0.005, Figure 1C; in TCGA: HR = 1.93, 95%CI = 1.39–2.68, log-rank *P* < 0.001, Figure 1D). In the multivariable cox regression model that the risk group was analyzed in combination with age, gender, smoking, and stage, similar correlation could be observed, indicating that the risk group based on the 12 genes was a robust and independent prognostic factor in different populations (Figures 3C,D). Since there are only stage I and II cases in GSE31210, we also test in validation datasets when only included stage I and II cases, which still demonstrated consistent result (Figures 3E,F).

The distribution of the expression level of these 12 genes and corresponding clinical factors in patients from GEO validation cohort and TCGA cohort were, respectively, exhibited in Figures 2B,C, which also showed consistent enrichment pattern with the discovery cohort. The expression of these 12 immune relevant genes showed positively correlation with the risk score. Meanwhile, the ROC analyses in the two validation groups also demonstrated the superiority of the risk score as a prognostic factor when considering the model's sensitivity and specificity, especially when combined the risk score with other factors together (AUCROC = 0.969, 95%CI = 0.63–0.76 in GEO external validation cohort, Figure 4B; AUCROC = 0.661, 95%CI = 0.60–0.76 in TCGA cohort, Figure 4C).

### Differentially Expressed Genes and Relevant Biological Pathways Associated to the 12-Genes Based Risk Score

**Figure 5:**
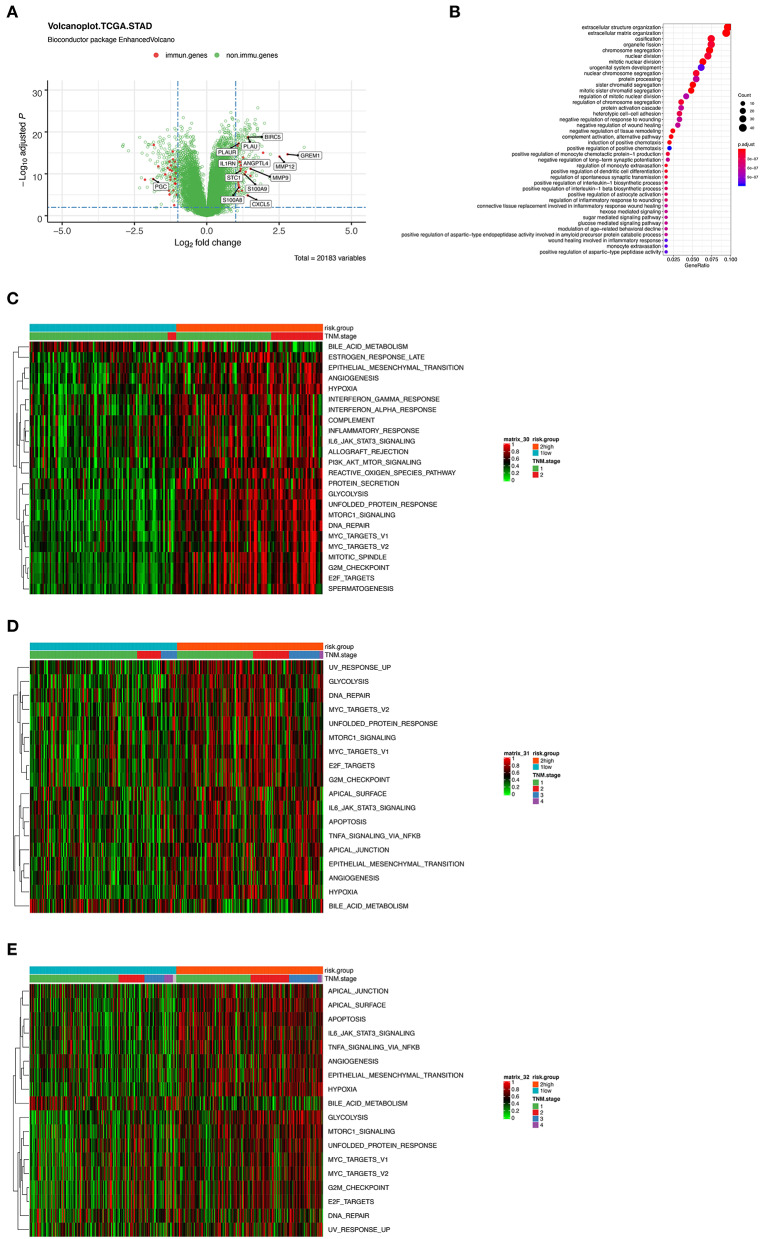
**(A)** Volcano plot presenting the differentially expressed genes (DEGs) between the high- and low-risk group. Red dots indicate immune-related genes and green indicate non-immune-related genes in GSE31210 cohort. **(B)** Gene Ontology (GO) functional enrichment analysis of the DEGs. **(C–E)** Heatmaps showing the enrichment score of a series of known gene-signatures by single sample gene set enrichment analyses in GSE31210 cohort **(C)**, GSE41271 and GSE50081 cohort **(D)**, and TCGA cohort **(E)**.

To further characterize the gene expression profiles of patients in high and low-risk score group, we performed differentially expressed gene (DEG) analyses. The result of DEG analyses was visualized in Figure 5A, where the important tumoral driver genes were annotated with their name. Gene Ontology (GO) functional enrichment demonstrated that expression alterations of these genes could activate not only tumor progression relevant pathways such as extracellular structure organization and nuclear division but also immune related processes like complement activation and regulation of monocyte extravasation (Figure 5B).

Furthermore, we performed ssGSEA using a series of known gene-signatures in each patient's expression profile. As shown in the heatmap (Figure 5C) where red region indicated the activation of corresponding pathways, we could observe both the up-regulation of several cancer related processes like hypoxia, epithelial mesenchymal transition, angiogenesis or PI3K-AKT-MTOR signaling, and immune-related pathway including interferon (IFN) γ response or complement cascade. The result of ssGSEA served as a supplementation of that of GO functional enrichment analysis. We inferred that complicated interaction exists among the 12 immune related genes and the mentioned pathways. Meanwhile, similar results were noticed in the two external validation cohorts (Figures 5D,E).

### Different Immune Infiltration Pattern

**Figure 6:**
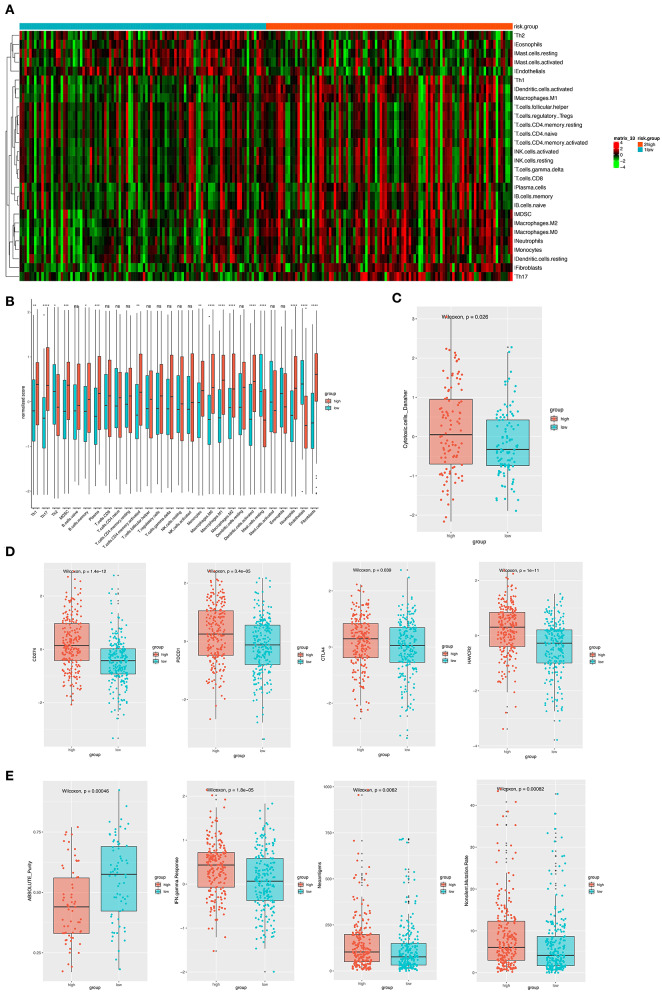
**(A)** The heatmap showing the infiltration pattern of 28 types of immune cell in patients from GSE31210 cohort. **(B)** The fraction of immune cells in high- and low-risk group in patients from GSE31210 cohort. Within each group, the thick lines in the boxes represents the median value. The bottom and top of the boxes are the 25th and 75th percentiles (interquartile range). The whiskers encompass 1.5 times the interquartile range. The statistical difference of two risk groups was compared through the Wilcoxon test. *p < 0.05, **p < 0.01, ***p < 0.001, and ****p < 0.0001. **(C)** Comparison of cytotoxic cells in the two risk groups. The statistical difference was compared through the Wilcoxon test. **(D)** The boxplots presenting the expression level of 4 immune checkpoint molecules (CD274, PDCD1, CTLA4, and HAVCR2) in high- and low-risk group from GSE31210 cohort. **(E)**. The boxplots presenting the level of 4 important immune-related parameters (absolute purity, IFN-gamma response, neoantigens, and non-silent mutation rate) in high- and low-risk group from GSE31210 cohort.

Considering the important role these 12 genes played in immune infiltration in the tumor microenvironment, we employed a reference microenvironment compendium that included 597 genes representing 28 immune cell subsets, to systematically characterize the immune infiltration pattern for patients from different risk group. We then estimated the relative abundance of the 28 immune cell populations in each sample using ssGSEA algorithm and compared the distribution of them across the high and low-risk score group. As exhibited in Figures 6A,B, the activation and recruitment of these immune cells were more frequently observed in patients from high-risk group, especially for Th1, Th2, Th17, MDSC, macrophages, which were mostly from innate immune response. Several other immune related parameters were also compared between the high and low-risk group. We found the up-regulation of cytotoxic cells, CD274 (PD-L1), PDCD1, CTLA-4, HAVCR2, IFN-γ, and so on in high-risk group, whereas opposite result was observed only in absolute tumor purity. Higher tumor mutation load was also found (Figures 6C–E). Therefore, we could propose that the patients with high-risk score had a distinct immune infiltrating pattern and might benefit more from immune-checkpoint inhibitor. These findings were validated in the GEO and TCGA external validation cohorts, where the results were in consistent with the findings above (Figures S1, S2).

## Discussion

During the past decades, advances in bioinformatics, including the widely used machine learning algorithms, have enabled researchers to analyze the large-cohort mRNA sequencing or microarray data from a completely new perspective (15, 30). In the present study, nearly 1,000 (*n* = 954) samples in total were enrolled, which to our knowledge is the largest cohorts used for establishing an immune relevant gene-signature-based prognostic scoring system in LUAD. To promise the consistency among different data sets, we only adopted the gene microarray data generated by Affymetrix Human Genome U133 Plus 2.0 Array, the most commonly used commercial microarrays platform in human cancer profiling (31), thus to some extent eliminating the intra-cohort heterogeneity. Cox regression analysis and random survival forest algorithm were conducted to select the most representative and robust survival associated genes from the large gene list, as the unfiltered high-dimension data might lead to a high risk of overfitting and limit the usability when the model was applied to another independent data set. Basing on these methods, we established a 12-gene-based risk score system for predicting prognosis for patients with LUAD individually.

According to the risk score value, LUAD patients were classified into high- and low-risk score groups. Considering the batch effect among different datasets, especially when different gene-expression evaluating approaches were used, like RNA sequencing and microarray, we used different cutoff value for each dataset included in this study to eliminate potential heterogeneity, as suggested by previous studies (4). In our study, we found that patients in high-risk group tend to have worse overall survival compared with those who have lower risk score. The prognostic value of this factor remained robust in the two external validation cohorts, while the independence of the factor was further confirmed by multivariable Cox analysis after adjusting for age, gender, smoking status, and TNM stage. Therefore, we suggest a more frequent clinical surveillance and follow up plan for those patients.

As exhibited in the heatmap, it is not difficult to notice the relative enrichment of the 12 signature genes in patients from high-risk group. It has been reported that some of these genes were involved in the formation and regulation of tumor microenvironment. For example, colony-stimulating factor 1 (CSF1) regulates macrophage differentiation via the CSF1 receptor (32). As we know, in several cancer type, tumor-associated macrophages (TAMs) and monocytes can promote immune-suppressive microenvironments to counteract immune evasion (33, 34). By secreting cytokines such as CSF1, tumors are able to recruit macrophages and support tumorigenesis by enhancing angiogenesis, tumor cell invasion, intravasation, and metastases via the secretion of metalloproteinases and inhibiting antitumor immunity by secreting immunosuppressive cytokines, such as IL10 (35–38), thus accelerating cancer development. In addition to its primary role in heme catabolism, HMOX-1 also modulates tumor microenvironment and impacts cancer progression through its anti-oxidative and anti-inflammatory functions (39). Meanwhile, its role in tumor cell migration capability has also been reported in lung cancer (40). VEGFA, an important member of vascular endothelial growth factor signaling pathway, mediates the tumoral angiogenesis, and results in the progression and metastasis of NSCLC (41, 42). Besides, Liu et al. (43) demonstrated that the co-expression of VEGFA and PD-L1 exhibited a worst overall survival in patients with resected LUAD, indicating the potential complicated interaction between VEGFA expression and immune checkpoint inhibitor therapy. The critical role TGFβ/BMP signaling pathway played in tumor cell growth, stemness, epithelial-mesenchymal transition, invasion, and migration in triple-negative breast cancer has also been published (44, 45). Although some of biological functions of the 12 genes have not been reported in LUAD, their role in tumorigenesis and cancer immunity still needs further investigation.

Recently, several novel multi-gene-based signatures in LUAD have been proposed. For instance, Wang and colleagues developed a 4-genes signature as an independent factor to classify LUAD patients with lymph nodes metastasis into low- and high-risk groups. The signature consisting of LDHA, ABAT, FAM117A, and INPP5J was generated by Least Absolute Shrinkage and Selection Operator (LASSO) algorithm and has been validated in an independent external dataset (46). Another research conducted by Li et al. (4) also reported a 16-genes signature by integrating several machine learning strategies. In addition, similar statistical method has been applied to miRNA and DNA methylation signature, as mentioned in the articles by Li et al. (47) and Wang et al. (48). However, none of these studies investigated the immune infiltration pattern associated with the risk group. Although several immune-associated prognostic biomarkers have been proposed in gastric cancer and melanoma (49, 50), the study concentrating on immune related signature is still absent in LUAD.

The importance of understanding the immunological landscape of LUAD in a large cohort should never be neglected, since this observation might reveal the underlying mechanism of response and resistance to specific immunomodulatory agents and help to guide a more effective and precise immunotherapy regimen (51). Therefore, here we used a combinational omics strategy to comprehensively evaluate the tumor microenvironment of the LUAD samples in high- and low-risk score group. Based on functional analysis, our findings suggested that the high-risk group were enriched for genes involved in extracellular matrix organization, angiogenesis, and epithelial mesenchymal transition, which are considered T-cell suppressive and immune-evasion assistant (52–54). Besides, we found the enrichment of hypoxia-relevant genes in patients in high-risk score group, indicating that the intratumoral hypoxia might modulate the tumoral immune response in many ways. Most evidence suggests that HIFs (hypoxia-inducible factors) exert a tumor-promoting effect by immunosuppression, including the attraction of myeloid-derived suppressor cells like macrophages and the inhibition of tumor-infiltrating cytotoxic T-lymphocyte activity (55), which was further supported by our findings that the infiltration level of macrophages were significantly increased in high-score group. We believe the combination of HIF inhibitors and immunotherapy would serve as a useful approach for clinical testing to further improve outcomes.

Next, using ssGSEA based immune signature, enrichment of both innate and adaptive immune cells like CD8+ T cells, B cells, macrophages, and NK cells was demonstrated in high-risk score group. This observation was further supported by the lower tumoral purity and higher intratumoral heterogeneity in such group. The phenomenon of the co-infiltration of cytotoxic cells and immune-suppressive cells including Treg, MDSCs, and tumor-associated macrophages has been reported in many cancer types (56–59) and likely reflects the negative feedback mechanism embedded in the systemic nature of immune regulation (60). Although the accumulation of CD8+ T cells has been identified by Bremnes et al. as a negative prognostic factor in LUAD (61), the presence of intratumoral CD8+ T cells and high PD-L1 expression in both tumor cells and stroma were strongly correlated to good responses to immune checkpoint inhibitors. Such tumors are defined as “hot” tumors with antitumor immunity and the “hot” immune infiltration has been demonstrated to be driven by higher tumor mutation load or neoantigen load (25, 62, 63). Meanwhile, considering the high-expression of several immune markers including PD-1 (PDCD1), PD-L1 (CD274), CTLA-4, and TGF-β, we could speculate that the patients in the high-risk score group might benefit more from anti-PD-1/PD-L1/CTLA-4 treatment. Taken into together, we suggested that the 12-gene signature and the corresponding risk score could serve as an indicator for predicting the response to immune therapy in LUAD.

The major limitation of this study is its retrospective nature. Moreover, since the expression data from the three GEO datasets involved in this study were all generated by the same microarray, some other immune-relevant genes might be missed. Meanwhile, because of the lack of expression data on patients receiving immune checkpoint inhibitors treatment and the unavailability of information regarding to immunotherapy outcome in TCGA and GEO database, we failed to verify our speculation that the 12-genes signature predicts response rate in patients with LUAD. Studies integrating RNA-sequence and clinical outcome for immune-checkpoint inhibitor treated LUAD patients is needed in the future. Further prospective and comprehensive studies are warranted to validate our findings, and experimental research on these genes may provide new insight into their biological functions.

## Conclusions

A risk score system was established based on a 12 immune relevant genes signature in LUAD, in which a high score was independently associated with significantly worse prognosis. When combined with several other clinical information, the scoring model could serve as a powerful and accurate tool for predicting survival of LUAD patients individually. Meanwhile, the tumors in high-risk score group tends to exhibit an up-regulated immune infiltration level, thus providing new insights into the interaction between infiltrating immune cells and tumor cells. Our findings were all validated in two independent external cohorts and may facilitate clinicians to choose the optimal therapeutic regimen for patients with LUAD more precisely.

## Data Availability Statement

Publicly available datasets were analyzed in this study, these can be found in The Cancer Genome Atlas (https://portal.gdc.cancer.gov/); the NCBI Gene Expression Omnibus (GSE31210, GSE41271, and GSE50081).

## Author Contributions

LX and GB: conception, design, provision of study materials or patients, collection and assembly of data, and data analysis and interpretation. HF: administrative support. All authors: manuscript writing and final approval of manuscript.

## Conflict of Interest

The authors declare that the research was conducted in the absence of any commercial or financial relationships that could be construed as a potential conflict of interest.

## Supplementary Material

Figures S1 **(A)** The heatmap showing the infiltration pattern of 28 types of immune cell in patients from GSE41271 and GSE50081 cohort. **(B)** The fraction of immune cells in high- and low-risk group in patients from GSE41271 and GSE50081 cohort. Within each group, the thick lines in the boxes represents the median value. The bottom and top of the boxes are the 25th and 75th percentiles (interquartile range). The whiskers encompass 1.5 times the interquartile range. The statistical difference of two risk groups was compared through the Wilcoxon test. ^*^*p* < 0.05, ^**^*p* < 0.01, ^***^*p* < 0.001, and ^****^*p* < 0.0001. **(C)** Comparison of cytotoxic cells in the two risk groups. The statistical difference was compared through the Wilcoxon test. **(D)** The boxplots presenting the expression level of 4 immune checkpoint molecules (CD274, PDCD1, CTLA4, and HAVCR2) in high- and low-risk group from GSE41271 and GSE50081 cohort.

Figures S2 **(A)** The heatmap showing the infiltration pattern of 28 types of immune cell in patients from TCGA cohort. **(B)** The fraction of immune cells in high- and low-risk group in patients from TCGA cohort. Within each group, the thick lines in the boxes represents the median value. The bottom and top of the boxes are the 25th and 75th percentiles (interquartile range). The whiskers encompass 1.5 times the interquartile range. The statistical difference of two risk groups was compared through the Wilcoxon test. ^*^*p* < 0.05, ^**^*p* < 0.01, ^***^*p* < 0.001, and ^****^*p* < 0.0001. **(C)** Comparison of cytotoxic cells in the two risk groups. The statistical difference was compared through the Wilcoxon test. **(D)** The boxplots presenting the expression level of 4 immune checkpoint molecules (CD274, PDCD1, CTLA4, and HAVCR2) in high- and low-risk group from TCGA.

Table S1 The baseline information, expression data, and corresponding risk group of lung adenocarcinoma patients in GSE31210.

Table S2 The baseline information, expression data, and corresponding risk group of lung adenocarcinoma patients in GSE41271 and GSE50081.

Table S3 The baseline information, expression data, and corresponding risk group of lung adenocarcinoma patients in TCGA database.

Table S4 The 336 immune-relevant genes selected by Cox regression.

Table S5 The 12 immune-relevant genes selected by random forest algorithm.

## Abbreviations

LUAD: lung adenocarcinoma
NSCLC: non-small cell lung cancer
GEO: Gene Expression Omnibus
TCGA: The Cancer Genome Atlas
OS: overall survival
HR: hazard ratio
DEGs: differentially expressed genes
GO: gene ontology
RSF-VH: random survival forest-variable hunting
VIMP: variable importance
AUCROC: area under receiver operating characteristic
IFN: interferon
ssGSEA: single sample gene set enrichment analysis
CSF1: colony-stimulating factor 1
TAMs: tumor-associated macrophages
LASSO: Least Absolute Shrinkage and Selection Operator.
